# Alteration of Light-Dependent Gene Regulation by the Absence of the RCO-1/RCM-1 Repressor Complex in the Fungus *Neurospora crassa*

**DOI:** 10.1371/journal.pone.0095069

**Published:** 2014-04-18

**Authors:** Carmen Ruger-Herreros, María del Mar Gil-Sánchez, Gencer Sancar, Michael Brunner, Luis M. Corrochano

**Affiliations:** 1 Department of Genetics, University of Seville, Sevilla, Spain; 2 Biochemistry Center, University of Heidelberg, Heidelberg, Germany; AIT Austrian Institute of Technology GmbH, Austria

## Abstract

The activation of transcription by light in the fungus *Neurospora crassa* requires the White Collar Complex (WCC), a photoreceptor and transcription factor complex. After light reception two WCCs interact and bind the promoters of light-regulated genes to activate transcription. This process is regulated by VVD, a small photoreceptor that disrupts the interaction between WCCs and leads to a reduction in transcription after long exposures to light. The *N. crassa* RCO-1/RCM-1 repressor complex is the homolog of the Tup1-Ssn6 repressor complex in yeast, and its absence modifies photoadaptation. We show that the absence of the RCO-1/RCM-1 repressor complex leads to several alterations in transcription that are gene-specific: an increase in the accumulation of mRNAs in the dark, a repression of transcription, and a derepression of transcription after long exposures to light. The absence of the RCO-1/RCM-1 repressor complex leads to lower VVD levels that are available for the regulation of the activity of the WCC. The reduction in the amount of VVD results in increased WCC binding to the promoters of light-regulated genes in the dark and after long exposures to light, leading to the modification of photoadaptation that has been observed in *rco-1* and *rcm-1* mutants. Our results show that the photoadaptation phenotype of mutants in the RCO-1/RCM-1 repressor complex is, at least in part, an indirect consequence of the reduction of *vvd* transcription, and the resulting modification in the regulation of transcription by the WCC.

## Introduction

The fungus *Neurospora crassa* uses light as an environmental signal to modulate several aspects of its biology. Blue light activates the biosynthesis of carotenoids, presumably for photoprotection, resets the circadian clock, and regulates the development of reproductive structures [1]. The first effect of the exposure to blue light is the activation of transcription through the activation of the White Collar Complex (WCC), a light-dependent transcription factor complex composed of WC-1 and WC-2 [2], [3]. These two proteins contain zinc-finger domains at their carboxyl ends for DNA binding and promoter recognition, and WC-1 has a LOV domain that allows the binding of a flavin chromophore for sensing blue light [4]–[7]. Upon light reception two WCCs interact through the LOV domains of each WC-1, and the resulting complex of WCCs binds to GATCGA sequence motifs in the promoters of light-regulated genes to activate transcription [3], [8]. The WCC interacts with several proteins in the dark (FRQ, the RNA helicase FRH, and the protein kinase C), and these interactions modify the localization and activity of the WCC. [9]–[14]. In addition, the stability, activity, and nuclear localization of the WCC are controlled by the protein kinase A, PKA [15].

Light exposure increases the accumulation of mRNAs from about 20% of *Neurospora* genes [3], [16]. Exposure to blue light leads to the accumulation of VVD, a small photoreceptor that disrupts the interaction between WCCs, leading to a reduction in light-dependent transcription after long exposures to light known as photoadaptation [3], [17], [18]. Photoadaptation is a general feature of light-dependent transcription and has been described in other fungi and plants [19]–[21].

The protein WC-1 is transiently phosphorylated after exposure to light, and the phosphorylation status of WC-1 correlates with photoadaptation [22]. Phosphorylation of the WCC reduces its capacity to bind to light-inducible promoters in vitro, and it has been proposed that light-dependent phosphorylation may play a critical role in photoadaptation [22].

Several *Neurospora* genes have been used to monitor gene regulation by development and light. Genes *con-10* and *con-6* are preferentially expressed during the development of conidia [23], and in vegetative mycelia after blue-light exposure [24]–[26]. The transcription factor FL is a major regulator of conidiation, and the transcription of *fl* is induced by development and light [27], [28]. Light also activates the expression of several genes that encode enzymes for the biosynthesis of carotenoids such as the *albino* genes (*al-1*, *al-2*, and *al-3*) and *cao-2* [29]–[31]. The activation of *con-10* and *con-6* transcription by light is transient; maximum mRNA accumulation is observed after 0.5–1 h of light exposure, but mRNA accumulation is reduced upon longer light exposures, a clear indication of photoadaptation [25]. A similar transient activation of transcription has been observed for *fl* [27] and for a large fraction of light-regulated genes that have been characterized by microarray hybridization [16].

RCO-1 and RCM-1, the *Neurospora* homologs of the corepressor complex subunits Tup1 and Ssn6 in yeast, play a role in photoadaptation [32]. The Tup1-Ssn6 complex in yeast represses gene expression through interactions with other protein partners [33], [34]. The Tup1-Ssn6 complex may repress gene expression through several non-exclusive mechanisms that include the direct inhibition of transcriptional activators, modification of local chromatin structure to prevent binding of transcriptional activators and initiation of RNA transcription, and direct inhibition of the transcription machinery [33], [34]. These mechanisms may regulate gene expression in a redundant manner [35].

RCO-1 and RCM-1 accumulate in *Neurospora* nuclei [32] and both proteins interact to form a repressor complex similar to that present in yeasts [36]. When exposed to five hours of light, the *rco-1* and *rcm-1* mutants show a sustained expression of light-induced genes [32] suggesting that the RCO-1/RCM-1 complex is involved in the repression of light-dependent gene transcription that leads to photoadaptation. RCO1-1 is essential for the regulation of the *Neurospora* clock and is required to maintain the chromatin structure of the *frq* locus [37]. It is possible that the RCO-1/RCM-1 repressor complex modifies the chromatin leading to an inhibition of the WCC-driven gene transcription, but the precise role of this complex in photoadaptation remains to be investigated. RCO-1 interacts transiently with the transcription factor CSP-1. Transcription of the gene *csp-1* is activated by light through the WCC and encodes a repressor controlled by phosphorylation after interaction with the RCO-1/RCM-1 complex. If the interaction of CSP-1 and the RCO-1/RCM-1 complex is compromised, CSP-1 no longer functions as a repressor. CSP-1 regulates several genes involved in different metabolic processes by direct interaction with their promoters. The DNA binding sites for CSP-1 and RCO-1 have been identified [36]. How CSP-1 and the RCO-1/RCM-1 complex interact to regulate gene transcription is unclear. Three additional mutant strains that displayed defects in photoadaptation have been identified using a genetic selection system. These mutations altered photoadaptation of only a specific group of genes, suggesting that regulation of photoadaptation is relatively gene specific. Unfortunately, none of the relevant genes have yet been identified [38].

In order to further characterize the mechanism of repression of light-dependent gene transcription by the RCO-1/RCM-1 complex we have characterized the effect of mutations in the components of the RCO-1/RCM-1 complex in the regulation by light of gene transcription and the kinetics of WCC binding to promoters.

## Results and Discussion

### Deregulation of gene transcription by light in mutants of the RCO-1/RCM-1 complex

**Figure 1:**
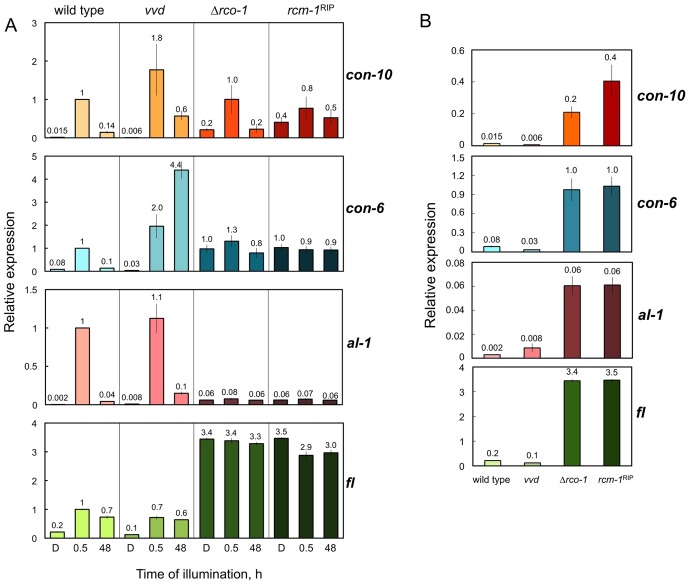
Photoactivation of light-inducible genes in the wild type and mutants. A. Quantitative RT-PCR experiments were performed to measure the relative accumulation of mRNAs in mycelia grown at 22°C for 48 h in the dark (D) or under continuous light (11 W m^-2^), or exposed to 30 minutes of light after 48 h of growth in the dark. The plots show the average and standard error of the mean of the relative mRNA accumulation in four independent experiments. The results from each PCR for each gene were normalized to the corresponding PCR for *tub-2* to correct for sampling errors and normalized to those obtained with the wild type after exposure to 30 min of light. B. A replot of the mRNA accumulation obtained in mycelia grown in the dark using a different scale.

Our previous work has shown that strains with mutations in *rco-1 and rcm-1* had an alteration in photoadaptation with a sustained mRNA accumulation for several genes after five hours of light, a phenotype that *rco-1* and *rcm-1* strains shared with *vvd* mutants [32]. In order to further characterize the role of the RCO-1/RCM-1 complex in the regulation of the activity of the WCC we measured the accumulation of mRNAs for several light-regulated genes after 30 min of light or after continuous illumination for two days. We used a strain with a deletion of the *rco-1* gene (Δ*rco-1*), a strain with a severely truncated version of *rcm-1* (*rcm-1^RIP^*) that retains some activity as the deletion of *rcm-1* was lethal [32], and the *N. crassa* wild-type strain and a strain with a mutation in *vvd* (*vvd* ^SS-692^) as controls. We hypothesized that mRNAs for light-regulated genes should accumulate in the strains without a functional RCO-1/RCM-1 complex after exposing mycelia to continuous light for two days. The mRNAs for *con-10*, *con-6*, *al-1*, and *fl* accumulated in mycelia of the wild-type or the *vvd* strain after 30 min of light, as measured by quantitative RT-PCR (Fig. 1A). Continuous illumination of wild-type mycelia reduced the amount of mRNAs for *con-10*, *con-6*, *al-1* and, to a lesser extent, *fl* due to photoadaptation. The amount of mRNAs for these genes after two days of light remained higher in the photoadaptation mutant *vvd* than in the wild type, as expected (Fig. 1A). The absence of the RCO-1/RCM-1 complex, however, altered the regulation by light of mRNA accumulation in an unexpected way. Light-dependent accumulation was observed for mRNAs of *con-10* in mycelia exposed to 30 min of light in the *rco-1* and *rcm-1* mutants, as previously published [32]. However, light-regulation was reduced for *con-6* in the *rcm-1* mutant, and the activation by light of *al-1*, and *fl* was completely abolished in strains without a functional RCO-1/RCM-1 complex (Fig.1A). We noticed that the amount of mRNAs for the genes under investigation increased in the dark in the *rco-1* and *rcm-1* mutants as compared to the wild-type or *vvd* strains (Fig. 1B), indicating that the RCO-1/RCM-1 complex acts as a gene repressor in the dark. Our results showed that in the absence of this repressor the activity of the WCC is affected such that it can activate gene transcription after light exposure for only some genes (*con-10* and partially *con-6*).

**Figure 2:**
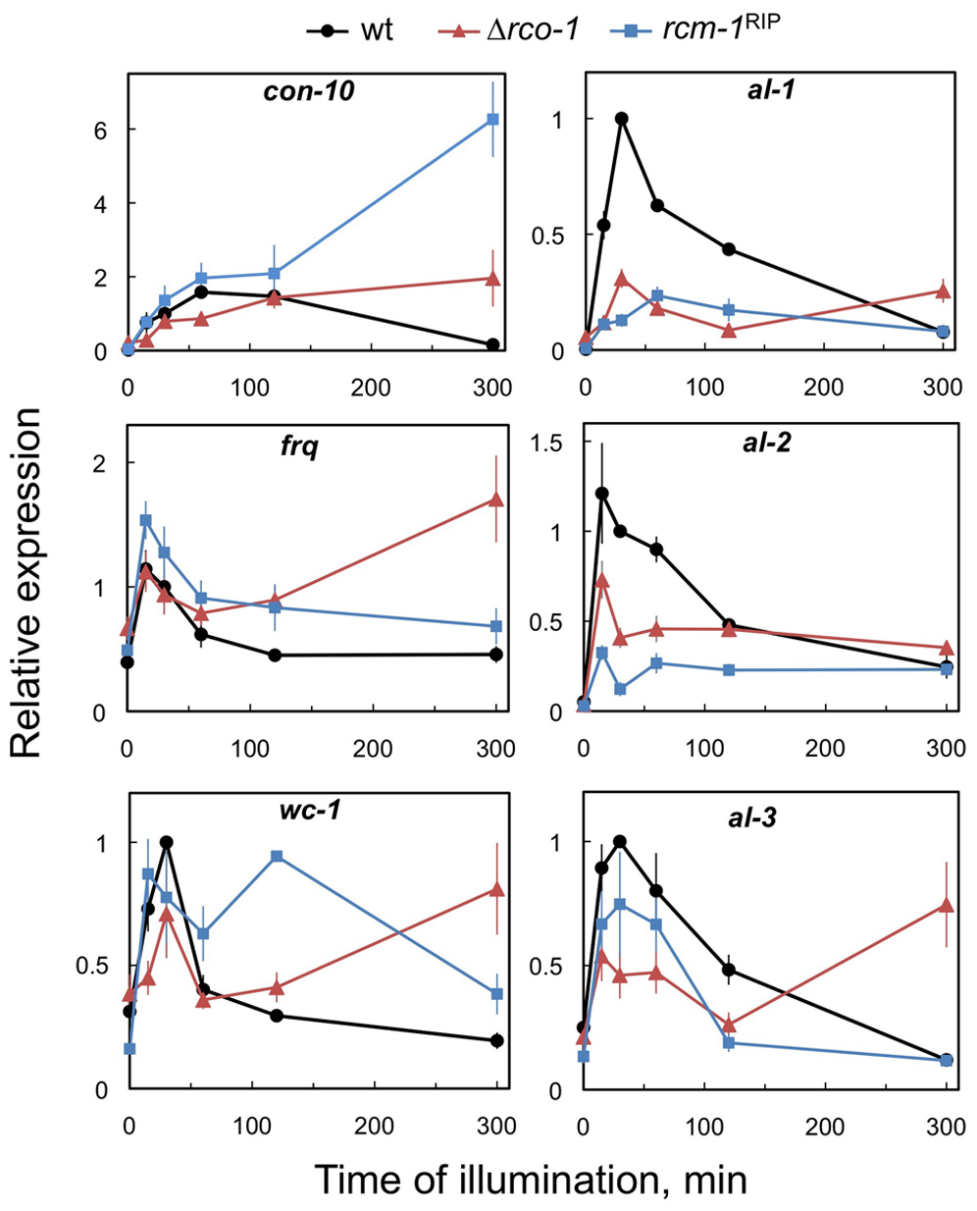
RCO-1 and RCM-1 regulate light-dependent gene transcription and photoadaptation. Quantitative RT-PCR experiments were performed to measure the relative accumulation of mRNA in mycelia of the wild type, a strain with a deletion of *rco-1* or a strain with the *rcm-1* ^RIP^ allele grown at 22°C for 48 h in the dark and exposed to white light (1 W/m^2^ blue light) for different times. The plots show the average and standard error of the mean of the relative mRNA accumulation in three independent experiments. The results from each PCR for each gene were normalized to the corresponding PCR for *tub-2* to correct for sampling errors and normalized to those obtained with the wild type after exposure to 30 min of light.

In order to characterize in more detail the alteration in light-dependent gene regulation in the absence of the RCO-1/RCM-1 complex we measured the accumulation of light-regulated mRNAs after exposures to light lasting up to 5 h. We measured light-dependent mRNA accumulation from genes involved in the biosynthesis of carotenoids, a process that is strictly controlled by blue light in vegetative mycelia [39]. We included in this characterization the *wc-1* gene, encoding a subunit of the WCC, the gene *frq*, encoding the major regulator of the circadian clock, and *con-10* as a control. These genes have been used routinely to assay the activation of gene transcription by light in *Neurospora* [16], [25], [40]. The activation of transcription in the wild-type strain triggered an increase in mRNA accumulation after short exposures to light, followed by a decrease in mRNA accumulation after longer light exposures due to photoadaptation (Fig. 2). The kinetics for light-dependent mRNA accumulation was specific for each gene: *frq* and *al-2* showed a very rapid activation of transcription with maximum mRNA accumulation observed after 5 min of light, compared with *con-10* that required 60 min of light to reach maximum mRNA accumulation (Fig. 2). The absence of the RCO-1/RCM-1 complex altered the kinetics of mRNA accumulation in different ways. Light-dependent mRNA accumulation was reduced for *al-1*, *al-2*, and *al-3*, but the effect was more marked for *al-1*, as we have shown earlier (Figs. 1A and 2). The opposite effect was observed for genes *con-10*, *frq*, and *wc-1*, as light-dependent mRNA accumulation increased with long exposures to light due to an alteration in photoadaptation, while light-regulation after short light exposures remained mostly unaltered (Fig. 2). The differences observed in the behavior of the *rco-1* and *rcm-1* mutants are probably due to the fact that we have used a strain with a deletion of *rco-1* and a strain with a severely truncated version of *rcm-1* that has retained some activity. Our results confirm the alteration in photoadaptation in the *rco-1* and *rcm-1* mutants described previously, and demonstrate that the RCO-1/RCM-1 complex acts as a gene repressor in the dark. The role of the RCO-1/RCM-1 complex in light-dependent gene regulation is gene-specific: for some genes (*con-10*, *frq*, and *wc-1*) it is required for proper photoadaptation, for others (*al-1*, *al-2*, and *al-3*) it is required to achieve full light-dependent gene regulation through the WCC. In yeast the Tup1-Ssn6 complex regulates transcription through the modification of local chromatin structure [33], [34], and RCO-1 is required to maintain the chromatin structure of the *frq* locus [37]. It is possible that the absence of the RCO-1/RCM-1 complex modifies the chromatin structure in a gene-specific way resulting in the alterations in light-dependent gene regulation that we have observed.

### The absence of the RCO-1/RCM-1 complex leads to a reduction of VVD after exposure to light

**Figure 3:**
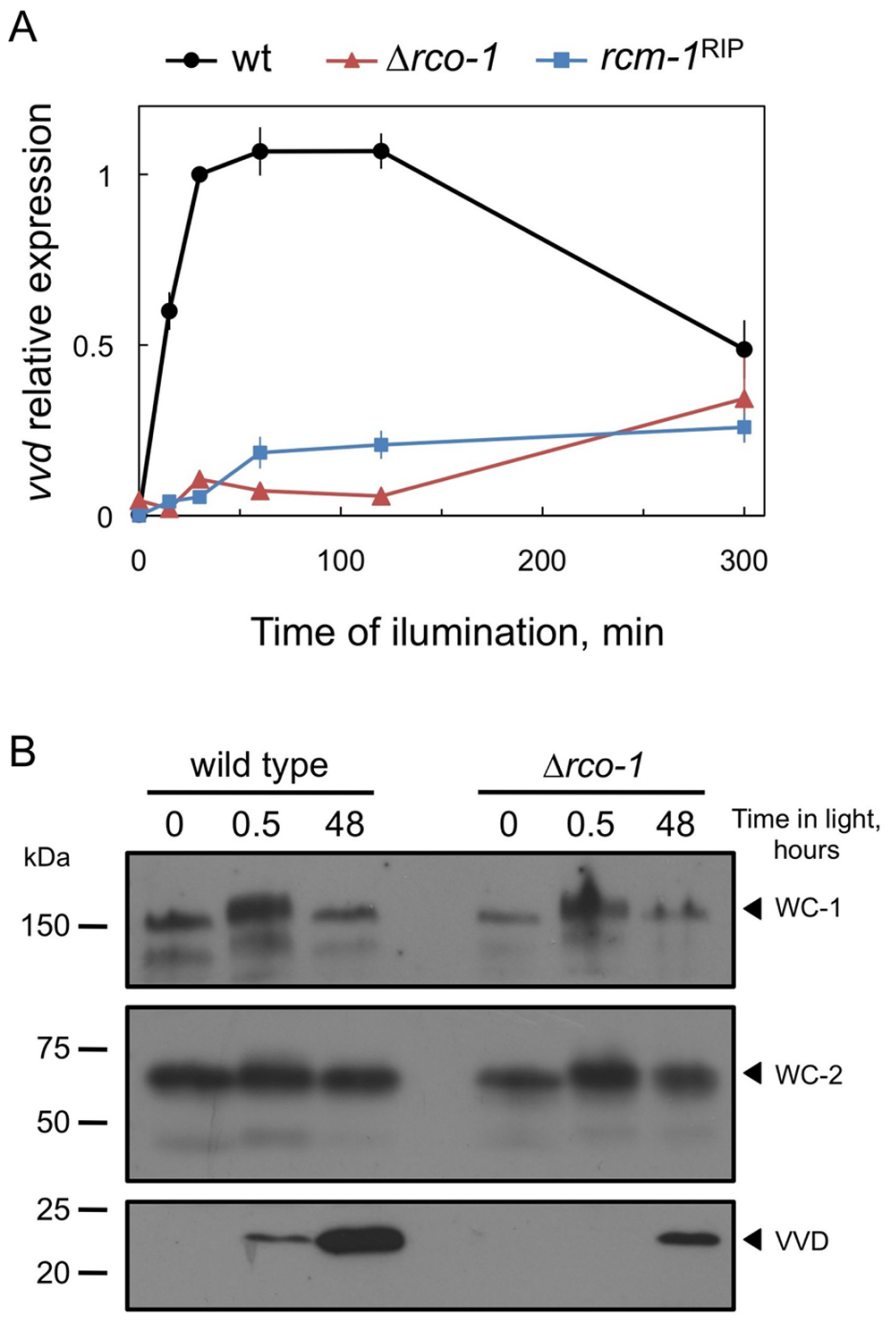
The RCO-1/RCM-1 complex is required for the accumulation of VVD after exposure to light. (A) The RCO-1/RCM-1 complex is required for the light-dependent mRNA accumulation of *vvd*. Quantitative RT-PCR experiments were performed to measure the relative accumulation of mRNA in mycelia of the wild type, a strain with a deletion of *rco-1* or a strain with the *rcm-1^RIP^* allele exposed to white light (1 W/m^2^ blue light) or kept in the dark. The plots show the average and standard error of the mean of the relative mRNA accumulation in three independent experiments. The results from each PCR for each gene were normalized to the corresponding PCR for *tub-2* to correct for sampling errors and normalized to those obtained with the wild type after exposure to 30 min of light. (B) A reduction in the amount of VVD in the Δ*rco-1* strain. Strains were ground on Vogel's minimal medium for 48 hours at 22°C in the darkness or under white light (11 W/m^2^). Then, samples grown in the dark were exposed for 30 minutes to light. Total protein extracts isolated from mycelia of the wild-type strain were separated by SDS–PAGE, and hybridized with anti-WC-1, anti-WC-2 and anti-VVD antibodies. 200 µg of proteins were loaded per lane. The bands that correspond to each protein are marked by an arrow, additional bands are due to WC-2 modification or to the non-specific binding of the WC-1 antibody. The experiment was repeated three times and a representative protein hybridization is shown.

The modification in the regulation by light of transcription in strains without a functional RCO-1/RCM-1 complex suggested that this repressor complex might modify the activity or the concentration of the WCC or VVD, the key regulatory elements. The RCO-1/RCM-1 complex interacts with the transcription factor CSP-1, and only RCM-1 was detected in immunoprecipitation experiments with a tagged version of RCO-1, ruling out a possible stable interaction between the RCO-1/RCM-1 complex and the WCC, at least under the conditions examined [36]. Since the RCO-1/RCM-1 complex is a general co-repressor, it is possible that the altered kinetics of light-dependent mRNA accumulation are a consequence of the missregulation of *wc-1* or *vvd* transcription. In the absence of the RCO-1/RCM-1 complex we only observed an increased accumulation of *wc-1* mRNAs after long-light exposures due to the alteration in photoadaptation (Fig. 2). The absence of the RCO-1/RCM-1 complex, however, reduced the light-dependent accumulation of *vvd* mRNA under all conditions that we tested (Fig. 3A). Our results show that the RCO-1/RCM-1 complex is required for the light-dependent accumulation of *vvd* mRNA.

We then assayed the amount of the two components of the WCC, WC-1 and WC-2, and VVD in the wild-type strain and in the Δ*rco-1* in cultures kept in the dark or exposed to light for 30 min or two days (Fig. 3B). No major differences were found in the amount and light-dependent phosphorylation of WC-1 and WC-2 between the wild-type strain and the Δ*rco-1* mutant, despite the increase in the amount of *wc-1* mRNA after long exposures to light (Fig. 2). The amount of VVD, however, was reduced in the Δ*rco-1* mutant as compared to the wild-type strain (Fig. 3B). Since the transcription of *vvd* is induced by light we did not observe VVD in the dark in the wild-type or mutant strains. After 30 min of light VVD was only detected in the wild-type mycelia. VVD was detected in mycelia of the Δ*rco-1* mutant after two days of light, but the amount in the mutant mycelia was less than in the wild-type strain (Fig. 3B). Our observations show that the absence of the RCO-1/RCM-1 complex leads to a reduction in the transcription of *vvd* and a subsequent reduction in the amount of VVD. Since VVD contributes to photoadaptation by disrupting the interaction between light-activated WCCs, our results suggest that the reduction in the amount of VVD that is available to inactivate the WCC dimers leads to an alteration in photoadaptation [3]. In accordance with this hypothesis the reduced amount of VVD in the Δ*rco-1* mutant should affect the kinetics of WCC binding to the promoters of light-regulated genes, which may cause the observed missregulation of light-dependent mRNA accumulation.

### The absence of the RCO-1/RCM-1 complex modifies the binding of the WCC to the promoters

**Figure 4:**
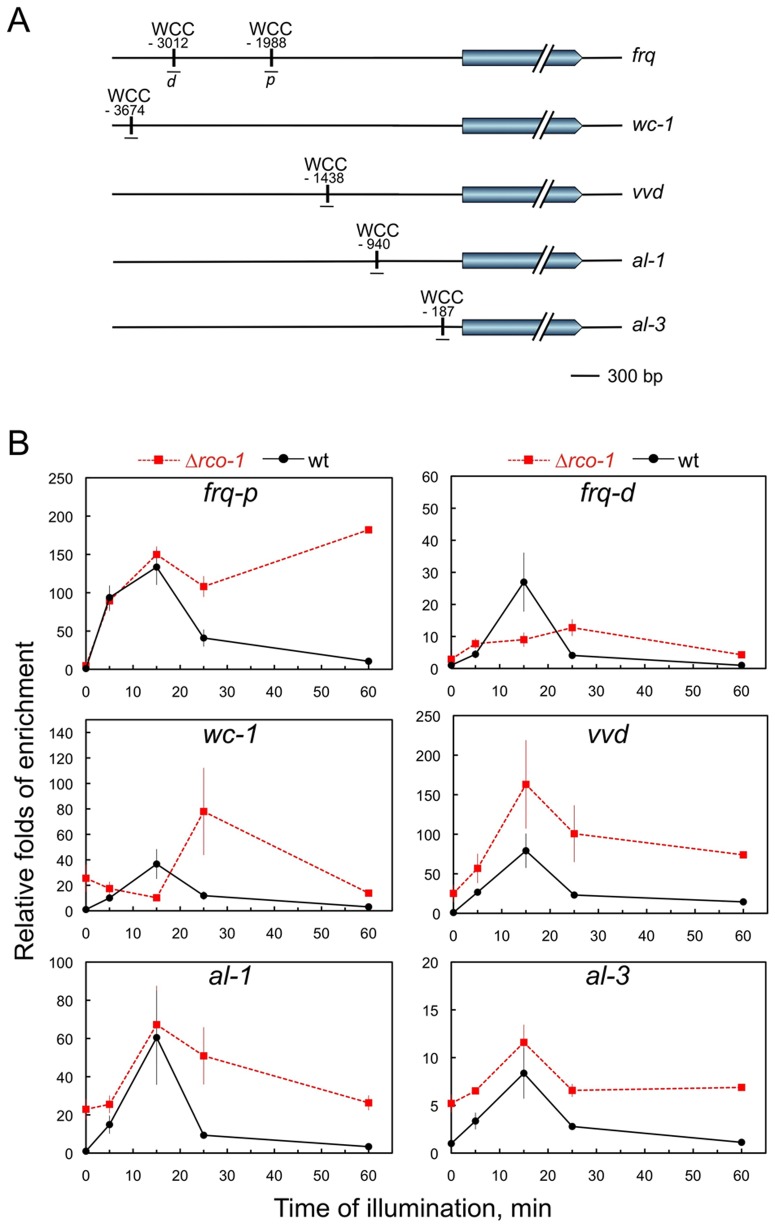
The absence of RCO-1 modifies the kinetics of WCC binding to the promoters of light-regulated genes. A. WCC binding sites in the promoters of several light-regulated genes. The position of the WCC binding sites in the promoters of *frq* (proximal site, *frq-p*; distal site, *frq-d*), *wc-1*, *vvd*, *al-1* and *al-3* are shown relative to the initiator ATG. B. Kinetics of WCC binding to the promoters measured by chromatin immunoprecipitation with an antibody against WC-2. Mycelia were grown for 48 hours at 30°C in the dark and then exposed for different times to light. Nuclei were extracted prior to each ChIP experiment. Quantitative PCR were performed to measure the relative accumulation of each DNA segment in immunoprecipitated samples and inputs. Each plot shows the average and standard error of the mean of DNA accumulation in three independent experiments. Results from each PCR for each gene were normalized to the corresponding PCR for 28S DNA to correct for sampling errors, and plotted relative to the amount obtained in the dark.

It has been shown previously that the WCC binds transiently to the promoters of *al-3*, *vvd*, *wc-1*, and the distal (*frq*-d) and proximal (*frq*-p) sites in the promoter of *frq* [7], [22], [27], [41]. To detect light-dependent binding of the WCC to the promoters of these light-regulated genes, chromatin immunoprecipitation with WC-2 antibodies followed by quantitative PCR (ChIP-PCR) was performed in wild-type and Δ*rco-1* strains exposed to light for different times or kept in the dark (Fig. 4). We observed that the absence of the RCO-1/RCM-1 complex resulted in increased WCC binding to DNA in the dark for all promoters, except for the *frq* promoter, when compared to the wild-type strain. A reduction in the amount of WC-2 binding to the clock region of the *frq* promoter (C box) in the dark has been observed for the *rco-1* mutant, suggesting a specific regulation of *frq* by the RCO-1/RCM-1 complex [37].

We observed maximum WCC binding to DNA after 15 min of light in both the wild-type and mutant strains for most promoters, but the WCC remained bound to most of the promoters after 60 min of light in the Δ*rco-1* mutant as compared to the wild-type strain (Fig. 4). The prolonged binding of the WCC could be due to the reduction in the amount of VVD observed in the Δ*rco-1* mutant.

### A model for the role of the RCO-1/RCM-1 complex in the regulation by light of transcription

Our results support an extension of our previous model for light-dependent gene regulation in *Neurospora* [3]: The RCO-1/RCM-1 complex acts as a gene repressor. Its absence leads to gene derepression (Fig. 1B), but also to attenuated light induction of some genes (Fig. 2). Our results suggest a functional competition of RCO-1/RCM-1-containing repressor complexes with activators/chromatin modifiers at the same promoters. Similarly, a dual role for RCO-1 in the regulation of *frq* transcription in the dark has been proposed, with RCO-1 suppressing WC-independent transcription and promoting WC binding to the promoter [37].

In the wild type, RCO-1/RCM-1 prevents transcription in the dark. Light promotes binding of the WCC and transcription activation, which may be associated with the release of the RCO-1/RCM-1 repressor. After prolonged exposure to light, newly synthesized VVD disrupts the interaction between WCC dimers so that they are released from the promoters and transcription is no longer activated.

In the absence of RCO-1/RCM-1-mediated repression unknown activators/chromatin modifiers support elevated transcription in the dark (see Fig. 1B). Light promotes binding of the WCC to its target sites, but the effect on transcription depends in a gene-specific manner on the RCO-1/RCM-1 complex. The absence of the RCO-1/RCM-1 complex from the promoters of *al-1* and *vvd* may result in chromatin changes as reported in yeast [42], [43] or in the *frq* promoter of *N. crassa* [37] that attenuate further activation of the promoter by the WCC in light. Hence, the lack of repression in the dark may functionally interfere with proper activation of these genes by the light-activated WCC. In accordance, the absence of the RCO-1/RCM-1 complex results in reduced accumulation of VVD after light exposure (Fig 3) despite prolonged promoter occupancy by the WCCs (Fig. 4). The RCO-1/RCM-1 complex should regulate *vvd* transcription in coordination with CSP-1, as both proteins were observed bound to the promoter of *vvd* [36]. The proposed alteration of chromatin in the *rco-1* or *rcm-1* mutants should be confirmed experimentally.

The light-dependent transcription of genes such as *con-10*, *frq* and *wc-1* was less affected by the absence of the RCO-1/RCM-1 complex, and the WCC can activate transcription of these genes after light exposure. Hence, factors (e.g. chromatin modifiers) that may interfere with WCC-dependent transcription activation are not recruited to these promoters. In addition, the reduced accumulation of VVD in RCO-1/RCM-1-deficient strains allows the prolonged binding of the WCC, resulting in sustained mRNA accumulation and the alteration in photoadaptation of these genes (*con-10*, *frq*, and *wc-1*, Fig. 2) [32]. The key role of VVD in photoadaptation [3], [17], [18], [44]–[46] suggests that the reduction in VVD leads to the alterations in photoadaptation reported for the *rco-1* and *rcm-1* mutants. However, our observations do not rule out the possibility that the absence of the RCO-1/RCM-1 complex leads to a direct alteration of photoadaptation.

In summary, our results reveal that the RCO-1/RCM-1-dependent repression in the dark is required for proper light induced transcription of some genes, including *vvd*. The reduced accumulation of VVD is in turn partially responsible for the altered light response of RCO-1/RCM-1-deficient strains.

## Materials and Methods

### Strains and culture conditions

We used the standard *Neurospora crassa* wild type strain 74-OR23-1VA (FGSC 2489, *matA*), and the mutant strains FGSC 7853 (*vvd^SS-692^ mat a*), FGSC 11372 (*rco-1^KO^ mat a*), and FGSC 10215 (*rcm-1* ^RIP^). *Neurospora* strains were obtained from the Fungal Genetics Stock Center (FGSC, http://www.fgsc.net). All strains were maintained by growth in Vogel's minimal media with 1.5% sucrose as carbon source. Strain manipulation and growth media preparation followed standard procedures and protocols [47]. See also, the *Neurospora* protocol guide (http://www.fgsc.net/Neurospora/NeurosporaProtocolGuide.htm).

### Light induction experiments

Cultures were grown and mycelia were illuminated for the times indicated to measure regulation of gene expression by light and to do chromatin immunoprecipitation (ChIP). Cultures were prepared by inoculating about 10^6^ conidia on liquid Vogel's minimal medium containing 0.2% Tween 80 as wetting agent. Liquid medium was used to prevent conidiation, and to allow the growth of *Neurospora* as submerged vegetative hyphae. The plates were incubated in the dark or in the light for 48 hours at 22°C. Samples incubated in the dark were exposed to white light provided by a set of fluorescent bulbs (containing 1 W/m^2^ of blue light). After light exposure mycelia were collected with the help of tweezers, dried on filter paper, wrapped in aluminum foil, frozen in liquid nitrogen, and stored at −80°C, unless otherwise indicated. Control cultures were kept in the dark prior to collection. All the manipulations in the dark were performed under red light.

### RNA isolation

*Neurospora* mycelia were disrupted by two 0.5-min pulses in a cell homogenizer (FastPrep-24, MP Biomedicals) with 1.5 g of zirconium beads (0.5 mm diameter) in 1.9-ml screw-cap tubes by using the RNeasy Plant Mini Kit (Qiagen) with the RLC buffer following the manufacturer procedure. The extracts in screw-cap tubes were clarified by centrifugation in a microcentrifuge (13,000 rpm) for 5 min prior to RNA purification. The RNA samples were treated with DNase I (USB) prior to use in RT-PCR experiments.

### Quantitative RT-PCR

The primers employed for quantitative RT-PCR are detailed in Table 1. Quantitative RT-PCR experiments were performed using one-step RT-PCR, in a LightCycler 480 II instrument (Roche) by using the One Step SYBR PrimeScript RT-PCR Kit (Takara Bio Inc.), 0.2 µM of each primer and 50 ng of RNA in a 10 µl reaction. The reaction consisted of 5 min at 42°C, followed by 10 s at 95°C, and then 40 cycles of DNA amplification (5 s at 95°C and 20 s at 60°C). After each PCR we performed melting curve analysis to show the specific amplification of single DNA segments and the absence of nonspecific amplified DNA. The fluorescent signal obtained for each gene was normalized to the corresponding fluorescent signal obtained with *tub-2* to correct for sampling errors. Expression data are shown relative to the wild type mycelia grown in the dark and illuminated for 30 minutes and are the average of at least three independent experiments.

**Table 1:** Oligonucleotides used in this work.

Gene	Forward Sequence (5′→3′)	Reverse Sequence (5′→3′)	Purpose
*con-10*	CAGCCACAGCGGAGGC	TTGGAGCAATTTCGCGC	RT-PCR
*con-6*	CGTCCTTGGCGGACACA	GGCGTTTTCAAGCACCTTCT	RT-PCR
*al-1*	TCCAATGTTTCCCCAACTACAAC	CGGTGGTGGGCGAGAA	RT-PCR
*al-2*	CGCTATCGCCTACCCCATT	CGACGAGGAAGCCTGTTTG	RT-PCR
*al-3*	CATCTCTTCCGCCGGTCTAG	ACCGAGGCCTTGCGTTTAC	RT-PCR
*frq*	AGCGCCGCCGATGAC	CCTTAAGCCGCTTGTTTTCG	RT-PCR
*wc-1*	TCATGCGCAAGGAATCCA	TCGCGATCTTGCCCTTTCTA	RT-PCR
*wc-2*	TTTGCACCCAATCCACAGAA	AGGGTCGAGCCATCATGAAC	RT-PCR
*vvd*	CGTCATGAGCTCTGATTCTG	GCTTCCGAGGCGTACACAA	RT-PCR
*fl*	GGCGATTCCCGCTACTGTT	TTGCAGGCCTTTCCCAAA	RT-PCR
p*frq-p*	CATCACTGCCCAGGTTCCA	GACGACGGCTGGCCAAT	ChIP
p*frq-d*	GTATCTTGAGCCTCCAGATCTCAAT	CCCGAGGCGTCCTGATG	ChIP
p*wc-1*	ATGGCCCAAAGCATTACAA	GCATTGCAAATTCGAAGAAA	ChIP
p*vvd*	CAGCACGAACAATTGAAACC	CCCGTTTTCTTTCCCTATCTG	ChIP
p*al-1*	CTCCATCGATCTGTCGAACC	GTCCCGGTCTTTCTGGTTG	ChIP
p*al-3*	CCCGCACGCTATGACGATA	ATAGCAAAGTGAGGTCGATTGCT	ChIP

### Total protein isolation and detection

Proteins were extracted from mycelia by previously described methods [48] using a modified lysis buffer (50 mM HEPES pH 7.4, 137 mM NaCl, 10% glycerol, 5 mM EDTA, 29.3 µM phenylmethyl-sulphonylfluoride (PMSF), 6.3 µM leupeptin, 4.4 µM pepstatin A and phosphatases inhibitors (PhosStop, Roche) when indicated. Total proteins were subjected to SDS-PAGE on 7.5% (30∶0.2) acrylamide-bisacrylamide gels and transferred to nitrocellulose membranes. Equal loading was confirmed by staining the hybridization membrane with Pounceau S solution. Membranes were hybridized with polyclonal antibodies against WC-1, WC-2 and VVD. Horseradish peroxidase-conjugated anti-rabbit IgG (BioRad) was used as secondary antibody. Antibody binding was observed by chemiluminescence (GE Healthcare).

### Preparation of nuclear and cytoplasmic protein extracts and chromatin immunoprecipitation (ChIP)

Nuclear and cytoplasmic protein extracts were obtained following published methods with minor modifications [7], [49], [50]. Wild-type *Neurospora crassa* was grown at 30°C during two days in the dark and then illuminated for different times. To allow protein-DNA crosslinking 1% formaldehyde (final concentration) was added to cultures after illuminations and they where incubated under shaking for 15 min. The crosslinking reaction was stopped by adding glycine (125 mM final concentration) and incubation under shaking for 5 min. Mycelia were collected and frozen on liquid nitrogen and stored at −80°C. Mycelial pads (5–6 g wet weight) were ground on liquid nitrogen and homogenized in 9 ml of buffer A (1 M sorbitol, 7% [w/v] Ficoll, 20% [v/v] glycerol, 5 mM magnesium acetate, 5 mM EGTA, 3 mM CaCl_2_, 3 mM dithiothreitol, 50 mM Tris/HCl, pH 7.5). The crude extract was filtered through a cheesecloth and 2 vol of buffer B (10% [v/v] glycerol, 5 mM magnesium acetate, 5 mM EGTA, 25 mM Tris-HCl, pH 7.5) were added to the filtrate while stirring. The homogenate was then layered onto 5 ml of buffer C (1∶1.7 mix of buffers A and B) in a Ultra-Clear tube (25×89 mm, Beckman), and centrifuged at 3000 g for 9 min at 4°C in a SW32Ti rotor (Beckman) to remove cell debris. Samples from the supernatant were taken (total cell lysate), frozen in liquid nitrogen and stored at −80°C. The remaining supernatant was then layered onto a 5 ml step gradient (1 M sucrose, 10% [v/v] glycerol, 5 mM magnesium acetate, 1 mM dithiothreitol, 25 mM Tris-HCl, pH 7.5) and centrifuged at 9400 g for 30 min at 4°C in a SW32Ti rotor. Aliquots from the supernatant were taken (cytoplasmic fraction), frozen in liquid nitrogen and stored at −80°C. The nuclear pellet was resuspended in buffer D (25% [v/v] glycerol, 5 mM magnesium acetate, 3 mM dithiothreitol, 0.1 mm EDTA, 25 mM Tris-HCl, pH 7.5), frozen in liquid nitrogen and stored at −80°C. All buffers contained protease inhibitors leupeptin (1 µM), pepstatin (1 µM), and PMSF (50 µM). Nuclear proteins were quantified by using Bradford assay and 2 mg of nuclei were subjected to chromatin immunoprecipitation. Nuclei were sonified for 8 minutes in a final volume of 0.5 ml of lysis buffer (50 mM HEPES [pH 7.5], 150 mM NaCl, 1 mM EDTA, 1% Triton X-100,0.1% deoxycholate, 0.1% SDS), (1 s on, 2 s off, 50% output, Sonoplus HD 2070/UW 2070 sonifier with the tip MS 72). The average DNA size obtained after sonication was 500–600 bp. Sonified samples were cleared at 15,000 g for 10 min at 4°C. Then, a fraction (1/10) of each supernatant was transferred to a new tube and marked as each “input” sample. Each remaining supernatant was precleared by the addition of 60 µl of salmon sperm DNA/protein A agarose mix (Millipore) and gentle rotation (4°, 60 min). After centrifugation (1 min, 13000 rpm), each supernatant was collected and divided in two; one half would be treated with the antibody, the “IP” sample, while the other half would remain as the “No-IP” control. Then, 50 µl of anti-WC-2 antibody (2 µg) [51] were added to about 200 µl from each “IP” supernatant, and incubated overnight at 4°C with gentle rotation. Then, 60 µl of salmon sperm DNA/protein A agarose mix (Millipore) were added to each sample (IP and No-IP), and incubated with gentle rotation (3 h, 4°C). The agarose beads were collected by centrifugation and cleaned twice with 1 ml of lysis buffer, once with LINDET buffer (0.01 M Tris-HCl pH 8, 1 mM EDTA, 0.25 M LiCl, 0.5% NP- 40, 1% sodium deoxycholate), and three times with TE buffer (10 mM Tris-HCl pH 7.5, 1 mM EDTA). DNA was eluted from the agarose beads by treating each sample with 320 µl of elution buffer (1% SDS, 0.1 M NaHCO_3_) incubating at 65° for 15 min and collected after centrifugation. The “input” samples were now included in the remaining protocol after increasing their volume by adding 270 µl of elution buffer. For protein-DNA de-crosslinking each sample received NaCl (0.2 M final concentration) and RNAse A (1.2 µg) and was incubated overnight at 65°C. Then, each sample was treated with proteinase K (10 µg) after addition of EDTA (10 mM final concentration), and Tris-HCl pH 8 (40 mM final concentration) (50°C, 1 h). Finally, DNA from each sample was purified using Wizard SV Gel and PCR Clean-up system (Promega). Protease inhibitors (1 mM PMSF, pepstatin 1 µg/ml, leupeptin 1 µg/ml) were included in all the buffers up to the DNA elution step.

### DNA Quantification by PCR

The amount of DNA in each ChIP sample was estimated after quantitative PCR using primers specific for the putative WCC binding sites in each promoter. Results from each PCR for each gene were normalized to the corresponding PCR for 28S DNA to correct for sampling errors and relativized to the dark point. Primer sequences are detailed in Table 1.
